# 

**Published:** 1906-12-01

**Authors:** 


**Notice to Advertisers and Subscribers.**

All Advertisements, Orders for Copies of The Hospital, and Business Communications should be addressed to **THE MANAGER (Not to the Editor),** The Hospital, 28 & 29 Southampton Street, Strand London, W.C.

The Cost of Subscription, post free, is as follows:—Per week, 3½d.; per quarter, 3s. 3d.; per half-year, 6s. 6d.; per annum, 13s. Foreign and Colonial:—Per annum, 19s. 6d., payable, in all cases, in advance.

### Christmas Presents for Nurses.

**1/=** By Post, 1/1

**NOTES ON PHARMACY AND DISPENSING FOR NURSES.**
New and Revised Edition. By C. J. S. THOMPSON.

**HOW TO BECOME A CERTIFIED MIDWIFE.**
By E. L. C. APPEL, M.B., B.S., B.Sc.Lond.

**SIMPLE INTRODUCTORY LESSONS IN MIDWIFERY.** By M. LOANE.

**THE NURSING OF SICK CHILDREN.** By Dr. BURNET.

**THE MODERN NURSING OF CONSUMPTION.** By Dr. JANE H. WALKER.

**SIMPLE SANITATION:** The Practical Application of the Laws of Health to Small Dwellings.
By M. LOANE. With an Introductory Chapter on Sanitary Legislation by Dr. A. MEARNS FRASER, Medical Officer of Health, Portsmouth.

**PRACTICAL HINTS ON DISTRICT NURSING.** By Miss AMY HUGHES.

**FEVERS AND INFECTIOUS DISEASES:** Their Nursing and Practical Management.
By WILLIAM HARDING, M.D. Ed., M.R.C.P. Lond., Author of "Mental Nursing," &c.

**1/6** By Post, 1/8

**SURGICAL INSTRUMENTS AND APPLIANCES USED IN OPERATIONS.** An Illustrated and Classified List, with Explanatory Notes.
By HAROLD BURROWS, M.B.Lond., B.S., F.R.C.S. New Edition, Revised and Enlarged.

**ART OF FEEDING THE INVALID.**
By a MEDICAL PRACTITIONER and a LADY PROFESSOR OF COOKERY. New and Popular Edition, with Special Chapter on Diet for Consumptives.

**THE CARE OF CHILDREN.** Practical Hints for Mothers and Nurses at Home and Abroad.
By ROBT. J. BLACKHAM, D.P.H.Lond., C.B. Revised and Enlarged Edition.

**LECTURES ON HOME NURSING FOR THE POOR.**
By a DISTRICT NURSE. Illustrated.

**OUTLINES OF ROUTINE IN DISTRICT NURSING.**
By M. LOANE, Superintendent of District Nurses, Portsmouth. 1/9 post free.

**OUTLINES OF INSANITY.** A Popular Treatise on the Salient Features of Insanity.
By FRANCIS H. WALMSLEY, M.D. Popular Edition.

**NURSING OF TROPICAL FEVERS.** By S. F. POLLARD, late Sister, Army Nursing Reserve.
The Work is based upon the Personal Experience of the Author Abroad.

**2/6** By Post, 2/9

**ELEMENTARY ANATOMY AND SURGERY FOR NURSES.**
By WILLIAM McADAM ECCLES, M.S.Lond., M.B., F.R.C.S., L.R.C.P. Profusely Illustrated.

**NURSING IN DISEASES OF THE THROAT, NOSE, AND EAR.**
By P. MACLEOD YEARSLEY, F.R.C.S.Eng., M.R.C.S., L.R.C.P.Lond. Illustrated.

**LECTURES UPON THE NURSING OF INFECTIOUS DISEASES.**

By F. J. WOOLLACOTT, M.A., M.D.

**MEDICAL ELECTRICITY AND LIGHT TREATMENT.**
A Practical Handbook for Nurses.
By KATE NEALE, Sister in Charge of the Actino-Therapeutic Department, Guy's Hospital. Profusely Illustrated.

**ELEMENTARY TREATISE ON THE LIGHT TREATMENT FOR NURSES.** By JAMES H. SEQUEIRA, M.D.Lond. Illustrated.

**HOSPITAL SISTERS AND THEIR DUTIES.**
By EVA C. E. LÜCKES, Matron, London Hospital. Third Edition, enlarged and partly re-written.

**TENDENCIES TO CONSUMPTION:** How to Counteract Them.
By J. D. E. MORTIMER, M.B.Lond.; M.R.C.S., L.S.A., Author of "Home Nursing of Sick Children."

**SPINAL CURVATURE AND AWKWARD DEPORTMENT:**
Their Causes and Prevention in Children.
By Dr. GEORGE MÜLLER, Professor of Medicine and Orthopædics, Berlin. English Edition, edited and adapted by RICHARD GREENE, F.R.C.P.Ed. With Plates and Illustrations.

*Complete Catalogue post free on application.*

**THE SCIENTIFIC PRESS, LTD.,**

**Nursing Publishers and Booksellers, 28 and 29 SOUTHAMPTON STREET, STRAND, LONDON, W.C.**

